# SIMS and Numerical Analysis of Asymmetrical Out-Diffusion of Hydrogen and Carbon in Cd*_x_*Zn_1−*x*_O:Eu Multilayer

**DOI:** 10.3390/ma17215240

**Published:** 2024-10-28

**Authors:** Zeinab Khosravizadeh, Anastasiia Lysak, Ewa Przeździecka, Rafał Jakieła

**Affiliations:** Institute of Physics, Polish Academy of Sciences, Aleja Lotnikow 32/46, 02-668 Warsaw, Poland

**Keywords:** Cd_*x*_Zn_1−*x*_O, diffusion, hydrogen, SIMS, semiconductor, annealing, FTCS method, activation energy

## Abstract

This study employs secondary ion mass spectrometry (SIMS) to investigate the diffusion behavior of hydrogen and carbon in a Cd*_x_*Zn_1−*x*_O:Eu multilayer at different annealing temperatures (500–900 °C). The SIMS results reveal a significant out-diffusion of these elements toward the surface and diffusion to the interface region. The diffusion flow rates are asymmetric and favor the interface direction. The depth profiles of diffused elements are fitted using the forward timecentered space (FTCS) iteration method. The activation energies are determined to be 0.35 ± 0.06 eV for hydrogen and 0.33 ± 0.09 eV for carbon, suggesting an interstitial mechanism in Cd_*x*_Zn_1−*x*_O. The results indicate that increasing the annealing temperatures leads to a significant decrease in impurity concentrations.

## 1. Introduction

The unique crystalline structures and electronic properties of ZnO (wurtzite structure) and CdO (rock salt structure) fundamentally contribute to their versatility in electronic and optical devices [1,2,3]. Alloying ZnO with CdO allows for flexible band gap modification [4,5], significantly enhancing its suitability for various applications, including optoelectronic devices and high-temperature/high-power electronics such as solar cells [6,7], fuel cells, photocatalysts [8], light detectors, laser sources, advanced oxide-based quantum devices [9], temperature sensors [10], gas sensors [11], UV photodetectors [12,13,14], and beyond [15]. Moreover, studies have explored how doping Europium (Eu) with CdO [16,17] and ZnO [18,19,20,21] significantly alters their structural and optoelectronic properties. For instance, Eu-doped ZnO nanopowders have been found to increase optical absorption and decrease the band gap, enabling control over emission intensity and wavelength based on the Eu doping percentage [22].

Hydrogen plays a crucial role in semiconductor technology, often appearing as a common impurity due to the challenges associated with its removal during crystal growth [23,24,25]. Shi et al. provided experimental evidence demonstrating by infrared (IR) spectroscopy that hydrogen is incorporated into as-grown ZnO crystals in an undetectable form [26]. This behavior is significant because residual impurities, such as hydrogen, can drastically affect the electrical and optical properties of materials in both industrial applications and material research [27,28,29]. For example, hydrogen plays a pivotal role in modulating conductivity in ZnO [30,31] and is similarly critical in other semiconductor-based devices like GaN and SiC [32,33]. In particular, hydrogen tends to become trapped in defects within the semiconductor structure, influencing material performance [34].

The diffusion behavior of hydrogen in ZnO has been explored through theoretical [35,36] and experimental methods, including SIMS [37,38,39,40,41,42,43,44,45]. Early studies by Mollwo (1954) and Thomas (1956) reported activation energies for hydrogen diffusion in ZnO of 1.12 eV and 0.91 eV, respectively [30,46]. More recent research has identified significantly lower activation energies than those reported by Mollwo and Thomas. For example, Ip et al. found an activation energy of 0.17 ± 0.12 eV [47], while Nickel observed values ranging from 0.17 to 0.37 eV for hydrogen diffusion in bulk ZnO [48]. These lower activation energies suggest that atomic hydrogen migrates via interstitial sites. Additionally, Wardle et al. reported activation energies in the range of 0.4–0.5 eV [35], indicating that trap-limited diffusion plays a significant role in hydrogen movement, even in undoped ZnO. This progression from earlier to more recent findings underscores the evolving understanding of hydrogen diffusion mechanisms and highlights the importance of interstitial migration.

Furthermore, theoretical studies on doped ZnO have shown that the activation energy for hydrogen diffusion can vary significantly depending on the doping elements. For example, in Li-doped ZnO, hydrogen diffusion can occur with an activation energy as low as 0.32 eV [49], while in Ag-doped ZnO, activation energies ranging from 0.3 to 1.0 eV have been reported [50]. Compared to the extensive research on hydrogen diffusion behavior in ZnO, studies on the effect of carbon in ZnO are limited. There are a few publications about the impact of carbon as a dopant on the electronic, crystallographic, optical, and magnetic properties of ZnO [51,52,53,54,55]. A notable discovery in the recent literature was the emergence of room-temperature ferromagnetic behavior by introducing carbon into this material [55,56]. These previous studies underscore the importance of further investigating the role of carbon in altering ZnO’s properties, particularly in thin film applications.

Achieving high-quality crystalline layers remains an enduring challenge due to the complexities of material processing and the potential introduction of impurities. These impurities can persist in as-grown samples and may also be influenced by annealing processes. Investigating the influence of annealing on the properties of ${\mathrm{Cd}}_{x}{\mathrm{Zn}}_{1-x}O$ is crucial for ensuring the material’s purity and optimizing its performance in optoelectronic applications. Depending on the atmospheric elements involved, the annealing process can potentially introduce additional impurities into the films, which can significantly impact the material’s properties [24,57].

In our study, we utilized high-purity $O_{2}$ gas during the post-growth annealing process, focusing on understanding the influence of annealing on the behavior of impurities in the CdZnO layer. Our SIMS depth profiles revealed that the dominant impurities in the ${\mathrm{Cd}}_{x}{\mathrm{Zn}}_{1-x}O$ thin films are hydrogen (H) and carbon (C), which primarily originate from the growth process itself. However, the existing literature provides limited studies on the effect of annealing on the properties of ZnO [58,59] and ${\mathrm{Cd}}_{x}{\mathrm{Zn}}_{1-x}O$ [5,9,25,57,60,61,62]. Therefore, it is critical to consider the role of atmospheric elements in such structures. One of the most essential properties of atoms in the solid is their ability to move through the lattice crystal at elevated temperatures, that is, the diffusion coefficient. Examining the diffusion parameter offers valuable insights into the material’s response to various annealing conditions, which can lead to advancing development in ${\mathrm{Cd}}_{x}{\mathrm{Zn}}_{1-x}O$-based electronic and optoelectronic devices.

The primary objective of this study is to investigate the diffusion profiles (i.e., the concentration versus depth profile curves) in both as-grown and annealed samples produced using the molecular beam epitaxy (MBE) method. To achieve this, we employed the secondary ion mass spectrometry technique, renowned for its precision in analyzing constituents, including dopants and matrix elements, in complex multilayered heterostructures [63]. SIMS is widely acknowledged for its effectiveness in estimating dopant depth profiles in semiconductors. In this study, concentration profiles of hydrogen and carbon from SIMS analysis are compared with those obtained from numerical simulations using a forward time-centered space (FTCS) method [64,65,66] implemented in a Python program.

The novelty of this work lies in applying the FTCS method for the first time to accurately model these complex behaviors (out-diffusion) of hydrogen and carbon profiles in CdZnO films. This method not only provides a more accurate fit of the experimental data, especially given the observed unusual diffusion profiles, but also offers a precise estimation of these profiles, which traditional diffusion models have failed to capture. By analyzing the SIMS profiles and comparing them with the results of FTCS simulations, we aim to estimate the diffusion coefficients of the mentioned elements and determine the temperature dependence of diffusivity using an Arrhenius plot.

This study provides new insights into hydrogen and carbon diffusion in ${\mathrm{Cd}}_{x}{\mathrm{Zn}}_{1-x}O$:Eu by demonstrating the effectiveness of the FTCS method in modeling complex diffusion profiles. Applying the FTCS method to these diffusion studies, especially for hydrogen and carbon, represents a novel approach that enhances our understanding of diffusion mechanisms in a CdZnO-based film. Furthermore, analyzing the diffusion characteristics provides a critical understanding of how the material reacts to different annealing processes. This deeper insight aids in improving the purity of ${\mathrm{Cd}}_{x}{\mathrm{Zn}}_{1-x}O$-based films and fosters progress in the enhancement of next-generation semiconductor technologies and optical devices.

## 2. Experiment and Methods

Thin films of ${\mathrm{Cd}}_{x}{\mathrm{Zn}}_{1-x}O$:Eu multilayer films (Cd contents range from 0.07% to 0.24%) were grown on Si substrates (SIEGERT WAFER GmbH, Aachen, Germany) using the MBE method (Riber Compact 21, Bezons, France) for intended optoelectronic study, as detailed in paper [67]. At first, the substrates were etched with buffered oxide for approximately 3 min, rinsed with distilled water, and then dried with nitrogen gas. Si substrates were degassed at 150 °C for 1 h in a load chamber and then the substrate temperature in the growth chamber was raised to 550 °C, and then lowered to the growth temperature of 360 °C, as measured by a thermocouple. A thin metallic Zn layer was deposited on the Si substrate for a few minutes to prevent oxidation. An RF cell was used to generate oxygen plasma, with the RF power set at 400 W and the $O_{2}$ gas flow rate at 3 sccm. High-purity Zn (6 N), Cd (6 N), and Eu (4 N) were used from Knudsen effusion cells. The base temperatures of the Cd and Zn effusion cells were maintained at 365 °C and 561 °C, respectively.

Before the growth process, the fluxes of Cd, Zn, and Eu were measured with a beam flux monitor of the Bayard–Alpert-type ionization gauge, showing approximately $1.33\times 10^{-4}$ Pa for Zn and $1.33\times 10^{-5}$ Pa for Cd. While Cd and Zn fluxes were kept constant, the Eu flux was varied. The ${\mathrm{Cd}}_{x}{\mathrm{Zn}}_{1-x}O$:Eu multilayer structures were capped with a CdO layer. The thickness of the CdZnO:Eu/ZnO:Eu sublayers was controlled by 2 and 5 min of deposition time, respectively, with the CdO cap layer deposited in 5 min. Different Eu concentrations were achieved by setting the europium effusion cell temperature to either 100 °C or 360 °C. Due to the low amount of Cd, the crystalline material [67] crystallized in a hexagonal structure [68]. The amounts of Cd and Eu and the thickness of the samples are presented in Table 1. The thickness of the films was measured using the DEKTAK 6M stylus profiler (Bruker, Karlsruhe, Germany) on craters that formed during SIMS measurements.

Rapid thermal processing (RTP) was performed in an Acuthermo AW610 system (Allwin21 Inc. (Morgan Hill, CA, USA)) to examine the effect of annealing on the concentration profiles of hydrogen and carbon. The samples underwent annealing at 500 °C for 20 min, and at 700 °C and 900 °C for 5 min each, under an oxygen atmosphere ($O_{2}$) [67]. A Hitachi SU-70 Scanning Electron Microscope (SEM) (Hitachi High-Tech Corporation, Tokyo, Japan) was used to capture the cross-sectional image of the ${\mathrm{Cd}}_{x}{\mathrm{Zn}}_{1-x}O$:Eu multilayer film. The SEM, equipped with a Thermo Scientific energy-dispersive X-ray (EDX) spectrometer (Thermo Fisher Scientific, Waltham, MA, USA), was also used to analyze the spatial elemental distribution in the films. EDX maps were collected under an accelerating voltage of 10 kV.

The SIMS measurements were conducted using a system equipped with an IMS6F magnetic sector instrument (Cameca, Gennevilliers, France). Samples were measured under the following conditions: a primary beam, Cesium ion (Cs), scanned an area of around 150 μm × 150 μm, and the secondary ions were collected from a central region of 60 microns in diameter. The negative ions were extracted at the primary beam energy of 14.5 keV and the positive ions at 5.5 keV, while the primary current was kept at 50 nA. For negative and positive secondary ions, the following species were measured: H−1, C−12, Si−28, Zn264O2−16, and OCs+16, SiCs+28, ZnCs+64, CdCs+114, and EuCs+153. The set of elements and reference ions, which were used for concentration estimation, was derived from [25]. In our work, the samples with high concentrations of H and C are considered for the study of the diffusion profiles. The background concentrations of H and C in the analyzed layers under the current measurement condition were at the level of $10^{18}\;{\mathrm{cm}}^{-3}$.

## 3. Results and Discussion

**Figure 1:**
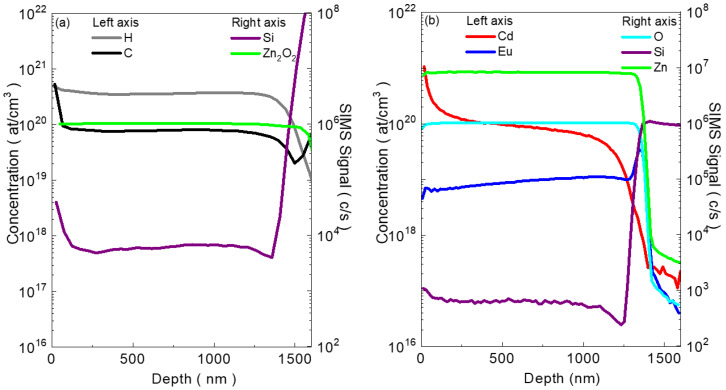
SIMS depth profiles of elements in the as-grown sample with high Eu content. Depth profiles for (**a**) hydrogen (H) and carbon (C) (left axis) and silicon (Si) and ${\mathrm{Zn}}_{2}O_{2}$ (right axis), and (**b**) cadmium (Cd) and europium (Eu) (left axis) and oxygen (O), silicon (Si), and zinc (Zn) (right axis). The concentration of Eu and Cd remained constant before and after annealing. The observed increase in the Cd signal at the surface is attributed to the CdO cap.

Figure 1a,b depict the SIMS depth profiles for elements collected in negative and positive secondary ion modes, respectively, for the sample with the highest amount of Eu. The data revealed that the concentrations of Eu and Cd remained constant throughout the annealing process. Due to the CdO cap, an increase in the Cd signal was observed at the surface, which was an artificial effect of SIMS measurement but did not affect our study of atmospheric elements.

**Figure 2:**
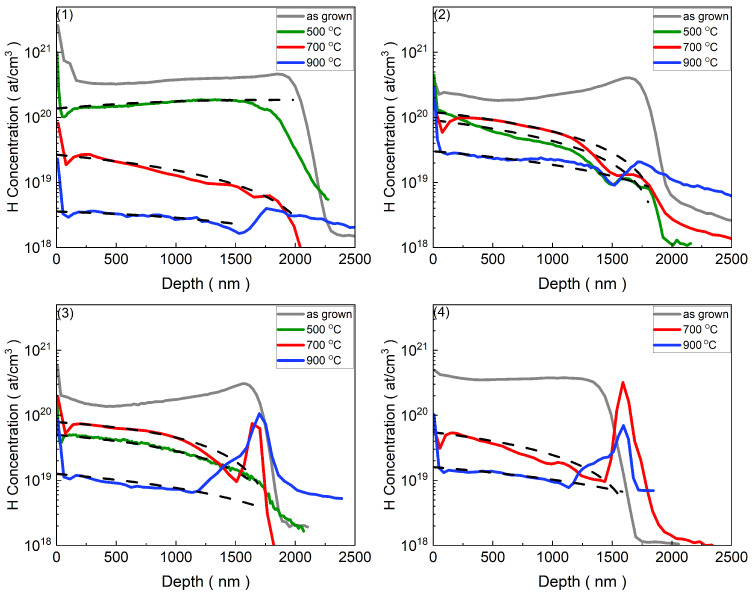
Hydrogen concentration profiles versus depth in ${\mathrm{Cd}}_{x}{\mathrm{Zn}}_{1-x}O$:Eu (Cd content <0.25%) under various annealing conditions. The profiles are shown for the as-grown sample (grey solid line), and after annealing at 500 °C for 20 min (green solid line), 700 °C for 5 min (red solid line), and 900 °C for 5 min (blue solid line). The dashed black lines represent the diffusion profiles estimated using the iteration method. Numbers (**1**)–(**4**) correspond to different samples, as listed in Table 1, where the Eu content increases progressively from sample 1 to sample 4.

**Figure 3:**
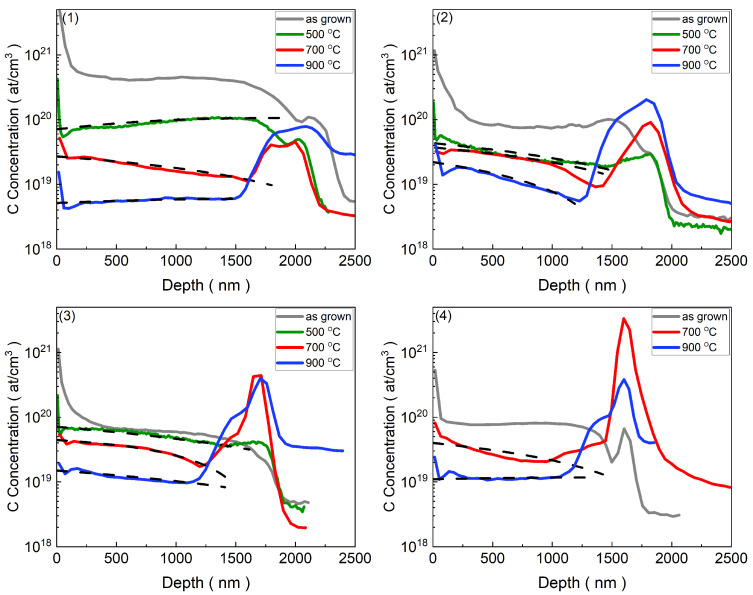
Carbon concentration profiles versus depth in ${\mathrm{Cd}}_{x}{\mathrm{Zn}}_{1-x}O$:Eu (Cd content <0.25%) under various annealing conditions. The profiles are shown for the as-grown sample (grey solid line), and after annealing at 500 °C for 20 min (green solid line), 700 °C for 5 min (red solid line), and 900 °C for 5 min (blue solid line). The dashed black lines represent the diffusion profiles estimated using the iteration method. Numbers (**1**)–(**4**) correspond to different samples, as listed in Table 1, where the Eu content increases progressively from sample 1 to sample 4.

Figure 2 and Figure 3 represent the distribution of hydrogen and carbon concentrations as a function of depth in the samples analyzed before and after annealing at various temperatures.

Our study found significant hydrogen and carbon impurities in the ${\mathrm{Cd}}_{x}{\mathrm{Zn}}_{1-x}O$:Eu films. These impurities originated primarily from the growth process rather than from post-growth annealing, as indicated by the SIMS depth profiles. The amounts of impurities in the films could be attributed to several potential sources in sub-optimal growth conditions, including the purity of source materials, vacuum quality, and the effectiveness of chamber maintenance. While it is challenging to eliminate all sources of impurities, minimizing their levels is essential for optimizing the material properties and performance of ${\mathrm{Cd}}_{x}{\mathrm{Zn}}_{1-x}O$ films. Moreover, it is crucial to understand the exact composition of residual gases and their potential impact on film quality.

Although hydrogen has the potential to form bubbles, which could impact diffusion, our study focuses on a crystalline ZnO-based film. The uniform crystal structure and lower defect density in such regions make bubble formation less likely than grain boundaries. In our results, due to a relatively low H level, the hydrogen concentration depth profile does not indicate bubble formation [33]. Upon annealing at a specific temperature and for a specific duration, impurity atoms diffused and formed a distinct diffusion profile. This diffusion process is governed by the diffusion coefficient, as outlined by Fick’s First Law (for one dimension):

(1) $$J=-D\frac{\partial C}{\partial z}$$

here, *J* represents the diffusivity flow, which is proportional to the concentration gradient of the dopant *C* and the diffusion coefficient *D* [69]. This relationship leads to Fick’s Second Law Equation (2), where *t* is the time variable.

(2) $$\frac{\partial C}{\partial t}=D\frac{\partial^{2}C}{\partial z^{2}}$$

The species are typically introduced into material from the ambient environment to study the diffusion of elements. The distribution of dopant atoms depends on the initial and boundary conditions. Fick’s law provides two solutions: one resulting from a constant source at the surface, described by the complementary error function (erfc); the second resulting from a limited source of diffusing species, defined by the Gaussian distribution function. If the distribution of diffusing particles deviates from the expected behavior dictated by the mentioned boundary conditions, iteration methods must be employed to solve this equation effectively.

For the aim of our experiment, samples with a high concentration of hydrogen and carbon in their as-grown samples were selected for measurement. As illustrated in Figure 2 and Figure 3, the concentration of hydrogen and carbon initially remained relatively constant and gradually decreased upon heating, indicating an out-diffusion profile. To better understand and characterize this behavior, we proposed a novel method to estimate the out-diffusion profile in the annealed samples. The observed out-diffusion profiles of the analyzed atoms underscored the need to utilize the iteration method to estimate the diffusion parameters. In addition, with an increase in temperature, the diffusion coefficient of the diffused elements follows the Arrhenius relation, which can be derived from the Boltzmann equation [70].

(3) $$D=D_{0}\exp\left(\frac{-E_{a}}{kT}\right)$$

where *D* is the diffusion coefficient, *T* is the temperature, $D_{0}$ is the pre-exponential factor, and $E_{a}$ represents the activation energy.

### 3.1. The Computational Method

The FTCS method [65,66], known for its effectiveness in numerically integrating ordinary diffusion equations, was applied in Python (Python 3.12.7). This code takes boundary conditions from SIMS depth profiles, including atomic concentrations at the surface, interface, and in the bulk of the layer of the as-grown sample, as input. Applying these parameters in the diffusion equation allows the estimation of the diffusion coefficient. The code utilized an iteration method by varying the diffusion coefficient to obtain the best-fitting profile with our experimental data, represented by the dashed lines in Figure 2 and Figure 3. Furthermore, the diffusion coefficient that best fits the data was extracted for each temperature to generate the Arrhenius plots. The FTCS equation is shown as follows:

(4) $$\frac{C_{j}^{n+1}-C_{j}^{n}}{\Delta t}=\frac{D_{j+1/2}\left(C_{j+1}^{n}-C_{j}^{n}\right)-D_{j-1/2}\left(C_{j}^{n}-C_{j-1}^{n}\right)}{{\left(\Delta z\right)}^{2}}$$

where *C* refers to the concentration of diffusing atoms, *D* represents the diffusion coefficient, *j* denotes the numbering of concentration points, and *n* denotes the numbering of iteration steps. In contrast, Δt and Δz represent the time and depth steps, respectively. The conditions set for simulation include the following:

(5) $$\Delta t\leq \frac{\Delta z^{2}}{2D_{j+1/2}}$$

### 3.2. Diffusion Coefficient of Hydrogen

The results obtained from the SIMS measurements of the hydrogen depth profiles (Figure 2) indicated a notable decrease in hydrogen concentration by approximately one order of magnitude in the samples annealed at 500 °C compared to the as-grown samples for the sample with the lowest amount of Eu (Figure 2(1)). Upon increasing the temperature to 700 °C and 900 °C, there was a significant decrease in hydrogen concentration for both samples with the lowest and highest amount of Eu (Figure 2(1),(4)). Ultimately, annealing at 900 °C reduced the number of hydrogen impurities by more than one order of magnitude for the high Eu-doped sample and by more than two orders of magnitude for the low Eu-doped sample.

**Figure 4:**
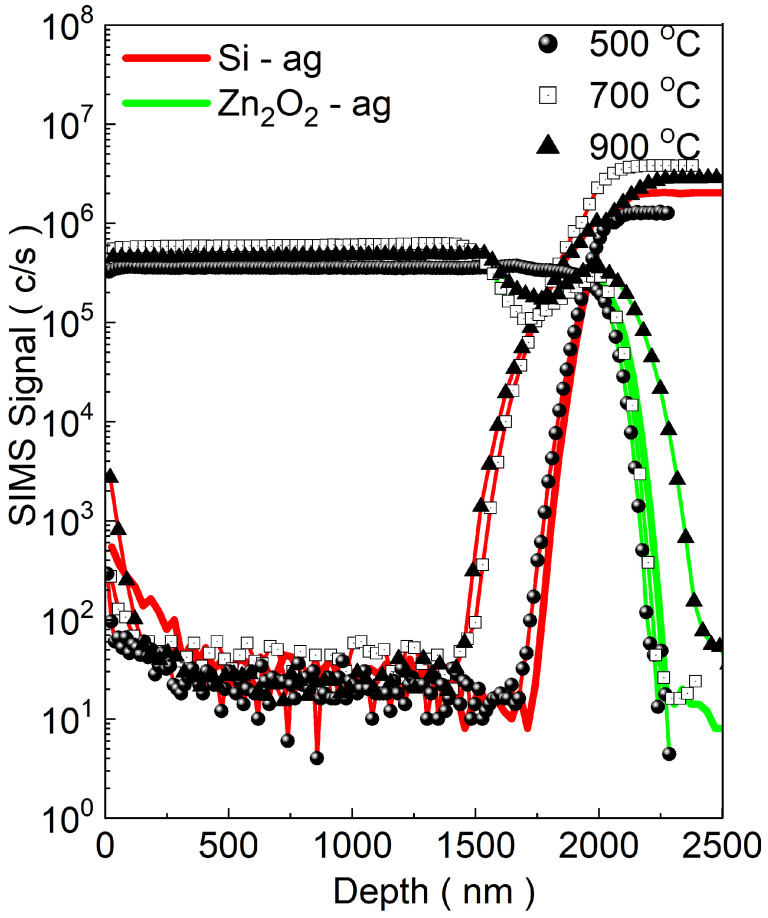
The SIMS depth profile of the reference’s signals of ${\mathrm{Zn}}_{2}O_{2}$ and silicon (Si). The profile labeled “ag” denotes the as-grown sample. The data reveal a sharp interface between ZnO and Si in the as-grown sample and at 500 °C. At higher annealing temperatures of 700 °C and 900 °C, Si is observed to penetrate deeper into the ZnO layer, indicating the formation of a $\mathrm{Si}O_{2}$ layer and changes in diffusion behavior.

Figure 4 illustrates an example of the SIMS depth profile for ${\mathrm{Zn}}_{2}O_{2}$ and Si signals. A sharp interface between ZnO and Si was observed in both the as-grown sample and the sample annealed at 500 °C. In contrast, for the samples annealed at 700 °C and 900 °C, Si penetrated deeper into ZnO, and a depletion of the ZnO signal near the interface was noted, suggesting the formation of a $\mathrm{Si}O_{2}$ layer. The peak in the ZnO signal at the interface with the Si substrate was likely due to a matrix effect, where the ${\mathrm{Zn}}_{2}O_{2}$ ion signal was influenced by the $\mathrm{Si}O_{2}$ layer. This $\mathrm{Si}O_{2}$ layer between ZnO and the Si substrate hindered most of the diffusion towards the substrate.

Due to the formation of the ${\mathrm{SiO}}_{2}$ layer, the concentration of hydrogen at the interface could not be treated in the same manner as the concentration in the ZnO layer. The observed increase in hydrogen’s SIMS signal in the ${\mathrm{SiO}}_{2}$ area was an artifact of the measurement, primarily due to the matrix effect. However, in the low Eu-doped sample annealed at 500 °C (Figure 2(1)), the hydrogen flow towards the surface exceeded that at the interface. This discrepancy may have stemmed from the absence of $\mathrm{Si}O_{2}$ layer formation at this temperature.

**Figure 5:**
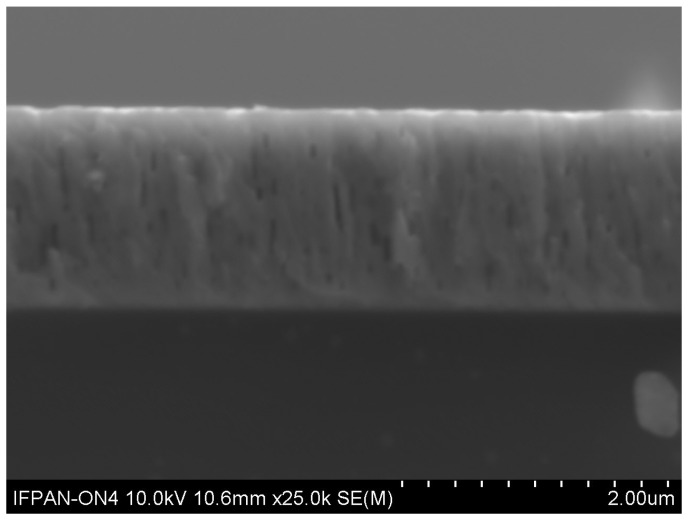
Cross-sectional SEM image of the ${\mathrm{Cd}}_{x}{\mathrm{Zn}}_{1-x}O$:Eu.

Figure 5 presents an example of a cross-sectional SEM image of the annealed sample, with a film thickness of 1.7 µm, after annealing at 900 °C. The observation confirmed that our thin films exhibit a generally uniform and well-defined crystalline structure.

**Figure 6:**
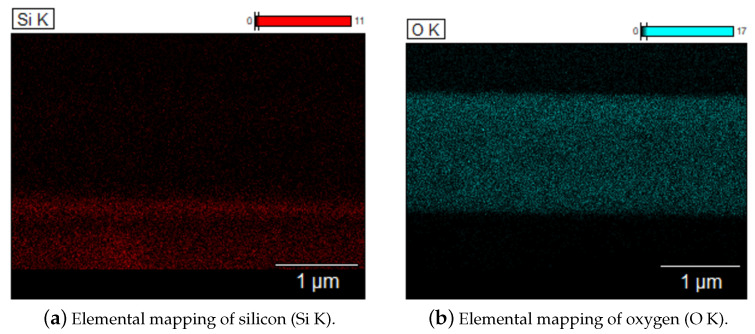
EDX elemental mapping of silicon (Si K-α line) (**a**) and oxygen (O K-α line) (**b**) from the cross-section of the ${\mathrm{Cd}}_{x}{\mathrm{Zn}}_{1-x}O$:Eu multilayer film. The maps confirm the formation of the ${\mathrm{SiO}}_{2}$ layer.

Figure 6a,b present EDX elemental maps for silicon (Si) and oxygen (O) from the cross-sectional analysis of the sample annealed at 900 °C. The oxygen distribution map delineates the extent of oxygen originating from the ZnCdO layer, while the silicon map corresponds to the silicon substrate. A thin shadow gap is observed within the silicon layer, attributed to the interface, and the presence of silicon in regions associated with oxygen suggests the formation of a ${\mathrm{SiO}}_{2}$ layer. The formation of this layer at the ZnO/Si interface becomes evident after annealing, especially at higher temperatures (700 °C and above). The thickness of the ${\mathrm{SiO}}_{2}$ layer, as estimated from the SIMS profile, is approximately 100 nm and it is primarily influenced by the temperature and duration time of the annealing process.

Moreover, the diffusion profile exhibited asymmetrical behavior. Notably, diffusion towards the interface region occurred faster than out-diffusion through the surface to the ambient environment. This asymmetrical behavior was likely due to the hydrogen surface concentration resulting from an equilibrium between the out-diffusion of hydrogen towards the ambient environment and the in-diffusion from the ambient environment into the material. As a result, the diffusion profiles deviated from the symmetrical shape, which should result from the symmetrical outflow of species on both sides: the surface and the interface. Consequently, the concentration of H atoms on the surface, determined by thermodynamic equilibrium, was incorporated as a boundary parameter in the iteration method.

**Figure 7:**
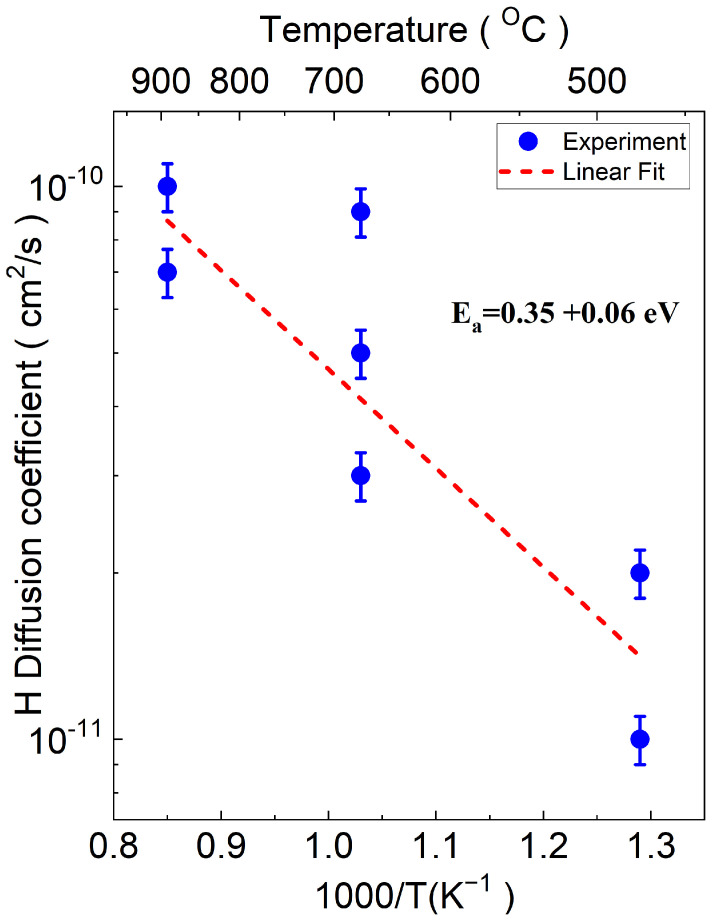
Arrhenius plot of the hydrogen diffusion coefficient (*D*) vs. reciprocal temperature (1000/T). The blue circles represent the experimental data points for different temperatures, while the red dashed line indicates the Arrhenius fitting curve obtained using Origin software. The spread of data points reflects variations in Eu concentration across different samples at varying temperatures. Some points may overlap due to similar diffusion values.

The Arrhenius plot of the diffusion coefficient of hydrogen is shown in Figure 7. The $E_{a}$ and $D_{o}$ for hydrogen were determined to be 0.35 eV and $2.9\times 10^{-9}\;{\mathrm{cm}}^{2}/s$, respectively. The estimated small activation energy suggests that hydrogen atoms predominantly occupy interstitial sites. A low activation energy is characteristic of interstitial diffusion, as this mechanism involves lower energy barriers compared to substitutional diffusion [36,71,72]. This typically occurs when solute atoms, which are smaller than the solvent atoms, occupy interstitial sites. The activation energy reported in the theoretical calculation based on the first-principles method (0.4–0.5 eV) [35,36] is in close agreement with the observed value in our study (0.35 eV). The discrepancy between the literature values (see the Introduction Section) and our findings may arise from several factors—material purity and the effects of Cd or Eu as dopants [49,50]—as well as potential interactions between hydrogen and carbon.

### 3.3. Diffusion Coefficient of Carbon

Figure 3 depicts the diffusion profiles of carbon. It was evident that the behavior of the carbon profiles in the as-grown and annealed samples closely resembled that of the hydrogen profiles. After annealing at 500 °C, 700 °C, and 900 °C, there was a notable decrease in the concentration of carbon in the sample with the lowest amount of Eu (Figure 3(1)). Increasing the annealing temperature resulted in a reduction in carbon impurities across all samples. Similar to hydrogen, the concentration of carbon at the interface could not be treated in the same manner as the concentration in the ZnO layer because of the formation of the ${\mathrm{SiO}}_{2}$ layer. Due to the matrix effect, carbon’s SIMS signal was artificially enhanced in the ${\mathrm{SiO}}_{2}$ area. However, at the annealing temperature of 500 °C, the flow of carbon toward the surface surpassed that toward the interface in the sample with the lowest amount of Eu. The same behavior was observed at 900 °C in both samples with the lowest and highest amounts of Eu (Figure 3(1),(4)), resulting in a consistent asymmetrical out-diffusion pattern similar to that observed in the hydrogen concentration profiles.

**Figure 8:**
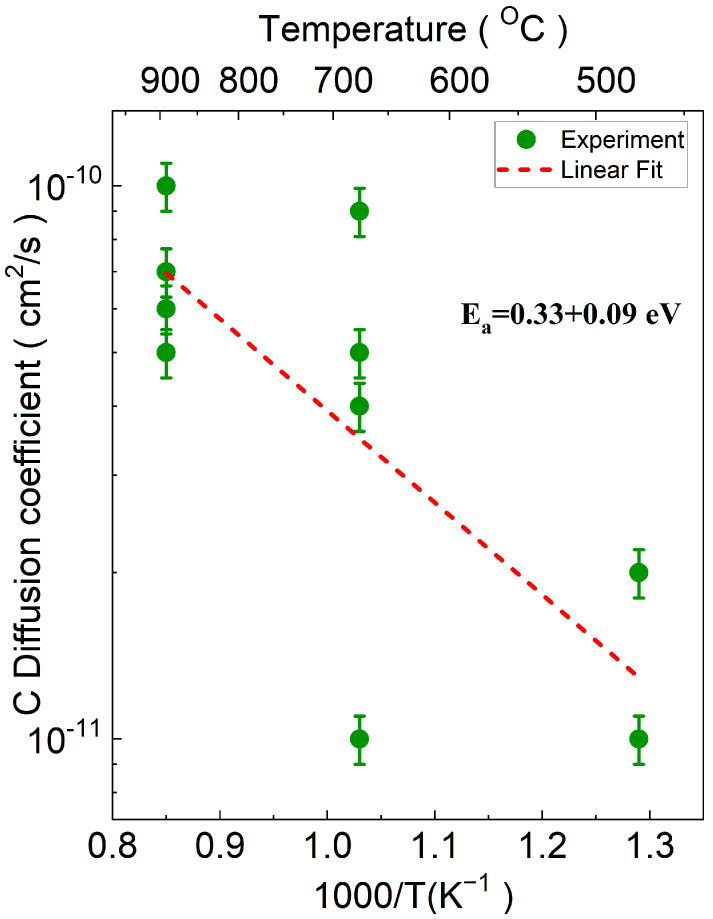
Arrhenius plot of the carbon diffusion coefficient (*D*) vs. reciprocal temperature (1000/T). The green circles represent the experimental data points for different temperatures, while the red dashed line indicates the Arrhenius fitting curve obtained using Origin software. The spread of data points reflects variations in Eu concentration across different samples at varying temperatures. Some points may overlap due to similar diffusion values.

Figure 8 presents the Arrhenius plot for the diffusion coefficient calculated from the data in Figure 3. Applying the Boltzmann equation (Equation (3)), which correlated the diffusion coefficient with temperature, enabled further analysis. The $E_{a}$ and $D_{o}$ for carbon diffusion were determined to be 0.33 eV and $1.8\times 10^{-9}\;{\mathrm{cm}}^{2}/s$, respectively. Similar to the results for hydrogen, the relatively low activation energy observed in our study implied that carbon also followed an interstitial diffusion mechanism. It could not be considered as grain boundary diffusion as the material showed a preferentially oriented crystalline structure [67]. Table 2 summarizes the diffusion coefficient parameters for both hydrogen and carbon, including their respective pre-exponential factors and activation energies.

In summary, the iterative method allowed the estimation of the diffusion coefficients for hydrogen and carbon, offering valuable insights into their diffusion characteristics. The results indicated that the low activation energy of atmospheric elements (hydrogen and carbon) led to relatively high diffusion coefficients, effectively removing them from the sample as the annealing temperature increased.

## 4. Conclusions

The hydrogen and carbon diffusion coefficients in ${\mathrm{Cd}}_{x}{\mathrm{Zn}}_{1-x}O$:Eu were estimated using an iterative method. The findings revealed a significant reduction in atmospheric element concentrations in the CdZnO layer. The low activation energy values suggested an interstitial diffusion mechanism for hydrogen and carbon in the material. Additionally, the formation of a $\mathrm{Si}O_{2}$ layer at the interface between ZnO and the Si substrate was identified as a cause of the asymmetric behavior of the depth profiles of analyzed impurities.

These results have important implications for designing and optimizing CdZnO-based devices. The low activation energies for hydrogen and carbon diffusion suggest that controlling the impurity concentrations during growth and annealing is crucial for tailoring the material properties. Specifically, understanding and mitigating the impact of interstitial diffusion could lead to improved device performance by reducing unwanted impurity effects. Future research should focus on exploring the impact of different annealing conditions and doping levels on the diffusion characteristics of atmospheric elements.

Additionally, investigating the formation and effects of interface layers, such as $\mathrm{Si}O_{2}$, on diffusion profiles could provide deeper insights into material behavior. These investigations will contribute to optimizing the fabrication processes and improving the performance and reliability of ${\mathrm{Cd}}_{x}{\mathrm{Zn}}_{1-x}O$-based devices in various applications, including optoelectronics and sensor technologies.

## Figures and Tables

**Table 1:** The amount of Cd and Eu and the thickness of the samples.

Sample	$x_{\mathrm{Cd}}$ $\;\left(\times 10^{-3}\right)$	Eu (${\mathrm{at}/\mathrm{cm}}^{3}$)	Thickness (µm)
1	1.4	$3\times 10^{16}$	2.00 ± 0.02
2	2.2	$4\times 10^{17}$	1.80 ± 0.02
3	0.7	$2\times 10^{18}$	1.70 ± 0.02
4	2.4	$1\times 10^{19}$	1.60 ± 0.02

**Table 2:** Diffusion coefficient parameters.

Element	Do $({\mathrm{cm}}^{2}/s)$	$E_{a}$ (eV)
Hydrogen	$\left(2.9\pm 0.5\right)\times 10^{-9}$	0.35 ± 0.06
Carbon	$\left(1.8\pm 0.3\right)\times 10^{-9}$	0.33 ± 0.09

## Data Availability

The data supporting the findings of this study are available from the corresponding author upon reasonable request. The code used in this study is not publicly available.

## Abbreviations

The following abbreviations are used in this manuscript:

SIMS	Secondary ion mass spectrometry
FTCS	Forward time-centered space
MBE	Molecular beam epitaxy
Eu	Europium
C	Carbon
H	Hydrogen
Cs	Cesium
SEM	Scanning Electron Microscope
EDX	Energy-Dispersive X-ray Spectroscopy
RTP	Rapid thermal processing
erfc	Complementary error function
ag	As-grown
